# Dairy fortification as a good option for dietary nutrition status improvement of 676 preschool children in China: A simulation study based on a cross-sectional diet survey (2018–2019)

**DOI:** 10.3389/fnut.2022.1081495

**Published:** 2022-12-08

**Authors:** Ye Ding, Fei Han, Zhencheng Xie, Genyuan Li, Yiding Zhuang, Jia Yin, Mingxian Fu, Jialu You, Zhixu Wang

**Affiliations:** ^1^Department of Maternal, Child and Adolescent Health, School of Public Health, Nanjing Medical University, Nanjing, China; ^2^Danone Open Science Research Center for Life-Transforming Nutrition, Shanghai, China

**Keywords:** Chinese preschool children, formulated milk powder, cow’s milk, soymilk, simulation

## Abstract

**Background:**

Chinese children are deficient in several essential nutrients due to poor dietary choices. Dairy products are a source of many under-consumed nutrients, but preschool children in China consume dairy products significantly less than the recommended level.

**Methods:**

From the cross-sectional dietary intake survey of infants and young children aged 0–6 years in China (2018–2019), preschool children (age: 3–6 years) (*n* = 676) were selected. The four-day dietary data (including 2 working days and 2 weekends) collected through an online diary with reference to the food atlas were used for analysis and simulation. In scenario 1, individual intake of liquid milk equivalents was substituted at a corresponding volume by soymilk, cow’s milk, or formulated milk powder for preschool children (FMP-PSC). In scenario 2, the amount of cow’s milk or FMP-PSC increased to ensure each child’s dairy intake reached the recommended amount (350 g/day). In both scenarios, the simulated nutrient intakes and nutritional inadequacy or surplus were compared to the survey’s actual baseline data.

**Results:**

It was suggested suggested that replacing dairy foods with FMP-PSC at matching volume is better than replacing them with soymilk or cow’s milk to increase the intake of DHA, calcium, iron, zinc, iodine, vitamin A, vitamin B_1_, vitamin B_3_, vitamin B_12_, vitamin C and vitamin D. Moreover, our results suggested that adding FMP-PSC to bring each child’s dairy intake to the recommended amount can bring the intakes of dietary fiber, DHA, calcium, iron, zinc, iodine, vitamin A, vitamin B_1_, vitamin B_3_, vitamin B_9_, vitamin B_12_, vitamin C and vitamin D more in line with the recommendations when compared with cow’s milk.

**Conclusion:**

Accurate nutrition information should be provided to the parents of preschool children so as to guide their scientific consumption of dairy products and the usage and addition of fortified dairy products can be encouraged as needed.

## Introduction

Preschool children (age: 3–6 years) are still undergoing rapid physical, psychological, and behavioral development, which increases their need for several nutrients, such as protein, polyunsaturated fatty acid, calcium, iron, zinc, vitamin A, B vitamins, vitamin C, and vitamin D (1, 2). Additionally, during this period, children’s self-awareness, curiosity, and imitation ability are also enhanced, and they are prone to developing various unhealthy eating and lifestyle habits, putting them at risk of nutritional imbalance (3, 4). Therefore, preschool children’s balanced nutrition should be provided by a balanced diet composed of a variety of foods, which should be further strengthened and consolidated to lay a foundation for healthy and good dietary behavior throughout their lives.

Although the status of child undernutrition in China has substantially improved due to economic growth, the problem of inadequacy or lack of dietary micronutrients has become prominent. According to China’s national dietary survey, children’s dietary mineral and vitamin intake remained inadequate in the past decade (5). Dairy products, as a good or excellent source of protein, calcium, phosphorus, magnesium, zinc, iodine, potassium, vitamin A, B vitamins, and vitamin D, play an important role in daily dietary recommendations in the dietary guidelines worldwide and are recognized as an ideal dietary composition to meet the growth and development needs of children and improve their nutritional status (6, 7). However, influenced by the conventional diet culture, dairy consumption is not a long-standing dietary practice in most Chinese households (8). In addition, regional economy, family income, nutrition knowledge, and other aspects also affect dairy consumption in China (9). Although the popular science propaganda to promote children’s dairy consumption persists, the dairy intake of children in China is far from the recommended level. According to survey data, 44.6% of children aged 2–5 years in China consumed dairy products, with the median amount being 106.7 ml/day (5). According to the scientific research report on dietary guidelines for Chinese residents (2021), a lack or low intake of dairy products in their diet is the direct cause of inadequate calcium, vitamin A and other nutrient intakes (6).

Nutritional fortification is one of the primary measures of nutritional intervention, which plays a role in improving the nutritional status of the population (10). Dairy products possess emulsification and hydrophilicity characteristics, making them suitable for nutritional fortification with fat-soluble or water-soluble nutrients (11). Micronutrient fortification of dairy products is permitted in most countries for preschool children, and the most common fortified dairy food is milk powder (12). In combination with the current status of children’s dietary nutrition and health in China, it is necessary to encourage children’s dietary diversity. At the same time, the dairy products consumed by children can be synergistically fortified with vitamins, minerals, and other nutrients according to their dietary intake (13). In addition, the importance of polyunsaturated fatty acids and dietary fiber in children’s nutrition and health has been confirmed (14, 15). Therefore, the use and addition of food ingredients or nutritional fortifiers beneficial to children’s growth and development can be encouraged in dairy products.

In order to study the impact of dairy products on the dietary nutrition status of children, Jia x et al. utilized data from the China Health and Nutrition Survey 2015 (2015 CHNS) to perform a simulation study on children aged 3–8 years (16). The results showed that increasing daily consumption to the recommended amount (300 g/day) would reduce nutritional gaps, and formulated milk powder for children aged ≥3 years is a good food source to facilitate children in meeting their nutritional needs (16). However, the contribution of dietary supplements was not considered in this study. In fact, dietary supplements have significantly contributed to the intake of some minerals and vitamins in China. A previous survey showed that 41.1% of children aged 3–6 years consumed dietary supplements in China, which was higher than that of children aged 7–12 years (17). Therefore, when assessing the impact of dairy products on preschool children’s dietary nutritional adequacy, both food consumption and dietary supplement use should be evaluated simultaneously. In addition, the 2022 Chinese balanced diet pagoda for preschool children recommends consuming dairy products daily, equivalent to 350–500 g/day of liquid milk (7). It is also necessary to increase the dairy product levels in the simulation.

Based on the data from a cross-sectional dietary intake survey of infants and young children aged 0–6 years in China (DSIYC, 2018–2019), this study aims to evaluate the impact of dairy products on the dietary nutritional status of 676 preschool children (age: 3–6 years) through two scenarios. In scenario 1, individual intake of liquid milk equivalents was simulated and substituted with a corresponding volume of soymilk, cow’s milk, or formulated milk powder for preschool children (FMP-PSC). In scenario 2, increasing the amount of cow’s milk or FMP-PSC to bring each child’s dairy intake to the recommended amount (350 g/day) was simulated. We hypothesized that the greatest improvements in nutrients of public health concern for preschool children would be observed when using FMP-PSC to simulate each scenario.

## Materials and methods

### Study population

The data of DSIYC from 2018 to 2019 were used in the analysis. First, two municipalities and 11 provinces, including Beijing, Shanghai, Guangdong, Sichuan, Yunnan, Fujian, Zhejiang, Jiangsu, Anhui, Hubei, Henan, Hebei, and Liaoning, were selected according to their geographical location, economic conditions, and annual live births. Then, the survey city was selected in accordance with the urban and rural areas of each region. Finally, according to the information provided by the maternal and child health center, preschool children were randomly recruited from each city. Based on the survey, we selected the effective dietary data of 676 preschool children aged 3–6 years, including 224 aged 3–4 years, 226 aged 4–5 years, and 226 aged 5–6 years.

### Dietary data collection

The dietary intake of preschool children was assessed through the use of an online diary with reference to the food atlas, which was developed by our research group using three visual reference systems, namely, regularly placed food portions, the two-dimensional background coordinates and common objects known in daily life (18). A food list composed of 323 types of food and drink was integrated into a 4-day online diary (including 2 working days and 2 weekends) to record food and drink intake. The data were collected by third parties (Danone Open Science Research Center and Taylor Nelson Sofres). Similar to previous research methods (19, 20), in face-to-face interviews, trained interviewers asked parents to report all foods and drinks, condiments, and dietary supplements consumed by the preschool children.

### Dietary data analysis

After the food was converted to the weight in its common state (i.e., 100% edible state and raw weight), the total daily intake of each kind of food was summed up. The individual components were recorded in the compound processed food, such as steamed stuffed buns, dumplings, wonton, steamed vermicelli roll, Chinese rice pudding, glutinous rice balls, hamburgers, pizza, and sandwiches. For example, in the chicken burger, bread, chicken, lettuce, and cheese were considered separately. The dairy products consumed were divided into 4 categories: liquid milk, milk powder, yogurt, and other dairy products (such as cheese, condensed milk, and milk tablets). The amounts of milk powder, yogurt, and other dairy foods were converted into their liquid milk equivalents in accordance with their protein composition. The total liquid milk equivalents were considered as the total intake of dairy products, and the proportion of preschool children who did not meet the recommended 350 g/day dairy intake in the 2022 balanced dietary pagoda for preschool children (Supplementary Figure 1) was calculated.

The energy and nutrient contents of each food were determined in accordance with the Chinese Food Composition Table (6th edition) (21). During the survey, the trade name of dietary supplements was recorded, and the nutrient content marked in the product manual was finally calculated together with the nutrient content from the dietary intake. According to the 2013 Chinese Dietary Reference Intakes (DRIs) (22), the intakes of most nutrients below the estimated average requirement (EAR) were perceived as inadequate, which included carbohydrate, protein, calcium, iron, zinc, iodine, vitamin A, vitamin B_1_, vitamin B_2_, vitamin B_3_, vitamin B_6_, vitamin B_9_, vitamin B_12_, vitamin C and vitamin D. Adequate intake (AI) was used to assess potassium inadequacy. Inadequate fiber intake was defined as the daily intake of <10 g/1,000 kcal energy. The upper limit of the acceptable macronutrient distribution ranges (AMDR) was applied to calculate the proportion of preschool children who consumed excessive fat.

### Modeling scenarios

In scenario 1, the individual intake of liquid milk equivalents was simulated to be replaced with soymilk (model 1), cow’s milk (model 2), and FMP-PSC (Aptamil) (model 3) at a matching volume. In scenario 2, cow’s milk (model 4) or FMP-PSC (model 5) was added so that each child’s dairy intake reached the recommended amount (350 g/day). In both scenarios, the nutrient intakes and the proportion of children with nutritional inadequacy or surplus after simulation were compared with the actual reported data. Nutritional composition per 100 g of soymilk, cow’s milk, and FMP-PSC used for simulation scenarios were shown in Supplementary Table 1.

### Statistical analyses

The normality of the continuous variables was tested and almost all dietary data were determined to show non-normal distribution; accordingly, they were represented by *P*_50_ (*P*_25_; *P*_75_). Categorical variables were expressed as frequency (*n*) and percentage (%). The participants were grouped by age, and the differences in dairy intake among different age groups were compared by Chi-square test or Kruskal Wallis H test according to the data type. Wilcoxon matched-pairs signed rank test and McNemar paired Chi-square test were applied to compare the differences in the nutrient intake and the changes in the proportion of preschool children with inadequate or excessive nutrient intake before and after modeling, respectively. Kruskal-Wallis H test or Mann-Whitney U test was applied to compare the energy changes of the different models. All data were statistically analyzed using the SPSS software package version 26.0 (IBM, New York, NY, USA). The results were considered to be statistically significant at *P* < 0.05.

## Results

### Dairy intakes before simulation

The distribution of dairy intakes before simulation (from DSIYC, 2018–2019) can be found in Table 1. Over 4 days, 92.01% of 676 preschool children aged 36–72 months consumed dairy foods. The dairy foods with the largest number among children were liquid milk, followed by milk powder. The median daily liquid milk equivalent was 174 g, with 88.31% of children falling short of the recommended amount (350 g/day).

**TABLE 1:** Dairy intakes before simulation.

Parameters	All (*n* = 676)	37∼48 months (*n* = 224)	49∼60 months (*n* = 226)	61∼72 months (*n* = 226)	*P*
Dairy consumption *N* (%)	622 (92.01%)	210 (93.75%)	207 (92.41%)	205 (91.52%)	0.473
Liquid milk *N* (%)	399 (59.02%)	122 (54.46%)	135 (59.73%)	142 (62.83%)	0.189
Milk powder *N* (%)	277 (40.98%)	112 (50.00%)	95 (42.04%)	70 (30.97%)	<0.001
Yogurt *N* (%)	236 (34.91%)	76 (33.93%)	81 (35.84%)	79 (34.96%)	0.913
Other dairy foods *N* (%)	118 (18.97%)	44 (19.64%)	36 (15.93%)	38 (16.81%)	0.556
Liquid milk equivalents (g/d)*	174.00 (84.61, 255.70)	196.72 (99.38, 280.84)	180.63 (100.00, 250.09)	149.38 (75.00, 244.25)	0.029
Below dairy recommendation *N* (%)	597 (88.31%)	193 (86.16%)	198 (88.39%)	206 (91.96%)	0.237

*Data were expressed in quartiles; *P*_50_ (*P*_25_, *P*_75_).

^△^*P* was assessed using the Chi-square test or Kruskal Wallis H test for the differences in dairy intake among different age groups.

Although there was no statistical difference in the proportion of consumers of liquid milk, yogurt, and other dairy products among age groups, there was a statistical difference in milk powder consumption among age groups. Specifically, as children aged, the proportion of milk powder consumed decreased. Although the proportion of children in all age groups who consumed less than the recommended amounts of dairy foods was high and there was no statistically significant difference among the groups, it is worth noting that, the amount of liquid milk equivalent consumed by children per day decreased with age, and the difference among all age groups was statistically significant.

### Total energy and macronutrient intakes after simulation

In scenario 1, after substituting the intake of liquid milk equivalents with soymilk (model 1), cow’s milk (model 2) and FMP-PSC (model 3) by matching volumes, compared with the reported data, the energy intake of children in all three models was significantly decreased, especially in the replacement of soymilk. The energy changes among the three models showed statistical differences (Table 2). There were similar changes in the intakes of carbohydrate and fat in each model. The protein intakes of all three models were comparable to the reported intake. The dietary fiber intake increased significantly after replacing with soymilk and FMP-PSC. The DHA intake findings showed that after FMP-PSC simulation, its value was significantly higher than that of other models (Table 3).

**TABLE 2:** Total energy intake after simulation (*n* = 676) [*P*_50_ (*P*_25_, *P*_75_)] (kcal/d).

Groups		Total energy	Changes of energy	*P* ^△^
Before simulation		993.99 (765.65, 1,256.99)	/	/
Scenario 1	Model 1	841.35 (657.73, 1,088.29)^a^	–70.28 (–187.38, –23.00)	<0.001
	Model 2	887.57 (690.99, 1,137.87)^bd^	–20.57 (–158.34, 0.00)	
	Model 3	911.78 (719.13, 1,162.83)^cef^	–0.16 (–129.96, 6.73)	
Scenario 2	Model 4	1,094.00 (874.92, 1,332.44)^g^	109.01 (65.27, 148.74)	<0.001
	Model 5	1,119.87 (906.53, 1,356.15)^hi^	139.02 (83.23, 189.68)	

Wilcoxon matched-pairs signed rank test was used to compare the differences in energy intake before and after modeling, respectively.

^a^Model 1 vs. Before simulation *P* < 0.05.

^b^Model 2 vs. Before simulation *P* < 0.05.

^c^Model 3 vs. Before simulation *P* < 0.05.

^d^Model 2 vs. Model 1 *P* < 0.05.

^e^Model 3 vs. Model 1 *P* < 0.05.

^f^Model 3 vs. Model 2 *P* < 0.05.

^g^Model 4 vs. Before simulation *P* < 0.05.

^h^Model 5 vs. Before simulation *P* < 0.05.

^i^Model 5 vs. Model 4 *P* < 0.05.

Model 1: The intake of liquid milk equivalents was replaced by soymilk at a matching volume.

Model 2: The intake of liquid milk equivalents was replaced by cow’s milk at a matching volume.

Model 3: The intake of liquid milk equivalents was replaced by FMP-PSC at a matching volume.

Model 4: The amount of cow’s milk was added to make the dairy intake of each child reach the recommended amount.

Model 5: The amount of FMP-PSC was added to make the dairy intake of each child reach the recommended amount.

^△^*P* was assessed using the Kruskal-Wallis H test (in Scenario 1) or Mann-Whitney U test (in Scenario 2) for changes of energy after simulation of different models.

**TABLE 3:** Macronutrient intakes after simulation (*n* = 676).

Macronutrients	Groups		*P*_50_ (*P*_25_, *P*_75_)	*N* (%)
Carbohydrate (g/d)	Before simulation		109.81 (83.05, 145.60)	388 (57.40%)
	Scenario 1	Model 1	100.18 (73.69, 133.98)^a^	444 (65.68%)^a^
		Model 2	104.02 (78.80, 137.97)^bd^	422 (62.43%)^bd^
		Model 3	109.98 (83.28, 144.39)^cef^	392 (57.99%)^ef^
	Scenario 2	Model 4	116.38 (89.74, 149.97)^g^	359 (53.11%)^g^
		Model 5	122.19 (95.56, 156.60)^hi^	330 (48.82%)^hi^
Protein (g/d)	Before simulation		32.65 (25.27, 43.05)	161 (23.82%)
	Scenario 1	Model 1	33.21 (25.69, 43.41)^a^	159 (23.52%)
		Model 2	33.21 (25.69, 43.41)^b^	159 (23.52%)
		Model 3	32.99 (25.57, 43.14)^cef^	161 (23.82%)
	Scenario 2	Model 4	38.46 (31.17, 47.53)^g^	65 (9.62%)^g^
		Model 5	38.34 (31.02, 47.40)^hi^	66 (9.76%)^h^
Fat (g/d)	Before simulation		37.87 (29.81, 48.28)	/
	Scenario 1	Model 1	34.31 (26.78, 43.96)^a^	/
		Model 2	37.29 (29.00, 47.34)^bd^	/
		Model 3	37.16 (28.90, 46.98)^cef^	/
	Scenario 2	Model 4	43.60 (36.50, 53.35)^g^	/
		Model 5	43.46 (36.31, 53.19)^hi^	/
Dietary fiber (g/d)	Before simulation		3.94 (2.79, 5.95)	668 (98.82%)
	Scenario 1	Model 1	6.03 (4.30, 8.36)^a^	581 (85.95%)^a^
		Model 2	3.93 (2.79, 5.95)^bd^	664 (98.22%)^d^
		Model 3	5.91 (4.24, 8.18)^cef^	636 (94.08%)^cef^
	Scenario 2	Model 4	3.94 (2.79, 5.95)^g^	671 (99.26%)
		Model 5	6.10 (4.70, 7.82)^hi^	658 (97.34%)^hi^
DHA (mg/d)	Before simulation		16.17 (8.89, 26.30)	/
	Scenario 1	Model 1	12.97 (7.36, 23.19)^a^	/
		Model 2	12.97 (7.32, 23.02)^b^	/
		Model 3	28.39 (17.27, 42.88)^cef^	/
	Scenario 2	Model 4	16.17 (8.89, 26.30)	/
		Model 5	32.31 (25.18, 40.76)^hi^	/

Wilcoxon matched-pairs signed rank test and McNemar paired Chi-square test were used to compare the differences in nutrient intake and the changes in the proportion of preschool children with inadequate nutrient intake before and after modeling, respectively.

^a^Model 1 vs. Before simulation *P* < 0.05.

^b^Model 2 vs. Before simulation *P* < 0.05.

^c^Model 3 vs. Before simulation *P* < 0.05.

^d^Model 2 vs. Model 1 *P* < 0.05.

^e^Model 3 vs. Model 1 *P* < 0.05.

^f^Model 3 vs. Model 2 *P* < 0.05.

^g^Model 4 vs. Before simulation *P* < 0.05.

^h^Model 5 vs. Before simulation *P* < 0.05.

^i^Model 5 vs. Model 4 *P* < 0.05.

Model 1: The intake of liquid milk equivalents was replaced by soymilk at a matching volume.

Model 2: The intake of liquid milk equivalents was replaced by cow’s milk at a matching volume.

Model 3: The intake of liquid milk equivalents was replaced by FMP-PSC at a matching volume.

Model 4: The amount of cow’s milk was added to make the dairy intake of each child reach the recommended amount.

Model 5: The amount of FMP-PSC was added to make the dairy intake of each child reach the recommended amount.

In scenario 2, after increasing the amount of cow’s milk (model 4) or FMP-PSC (model 5) to make the dairy intake of each child reach the recommended amount, compared to the reported intake, the energy intake of children in both models was significantly increased. The differences in energy between the two models were statistically significant (Table 2). The changes in carbohydrate, protein and fat intakes were shown to be similar to changes in energy consumption in both models, and the proportion of children with inadequate carbohydrate and protein intake decreased significantly. The intake of dietary fiber and DHA increased significantly after the addition of FMP-PSC, and their values were significantly higher than those after the addition of cow’s milk (Table 3).

The intakes of carbohydrates, protein, fat, dietary fiber, and DHA by different age groups after simulation were shown in Supplementary Table 2, and the changes in these nutrients were similar to those of the entire age group.

Table 4 displayed the surplus energy contribution from fat (%E) of the 49–60-month-old and the 61–72-month-old groups. The energy contribution from fat increased slightly in all five models when compared with the reported intake in both age groups. In both scenario 1 and scenario 2, cow’s milk simulation (model 2 and model 4) showed that the proportion of children with excessive energy contribution from fat was higher than that of other simulation groups.

**TABLE 4:** Energy contribution from fat after simulation.

Nutrient	Groups		49–60 months (*n* = 226)		61–72 months (*n* = 226)	
			*P*_50_ (*P*_25_, *P*_75_)	*N* (%)	*P*_50_ (*P*_25_, *P*_75_)	*N* (%)
Fat (%E)	Before simulation		37.68 (32.23, 41.77)	189 (83.63%)	35.78 (30.08, 40.80)	170 (75.22%)
	Scenario 1	Model 1	38.66 (34.74, 42.61)	208 (92.04%)^a^	37.64 (33.36, 42.65)^a^	196 (86.73%)
		Model 2	39.78 (36.05, 43.36)^bd^	210 (92.92%)^b^	38.71 (34.23, 43.63)^bd^	204 (90.27%)
		Model 3	38.36 (34.92, 42.37)^ef^	208 (92.04%)^c^	37.50 (33.54, 42.02)^cef^	198 (87.61%)
	Scenario 2	Model 4	39.71 (34.47, 43.05)^g^	205 (90.71%)^g^	38.20 (32.98, 43.15)^g^	200 (88.50%)^g^
		Model 5	38.39 (33.47, 41.65)^hi^	197 (87.17%)^hi^	36.92 (32.28, 40.92)^hi^	194 (85.84%)^hi^

The upper limit of the acceptable macronutrient distribution ranges was used to calculate the proportion of preschool children with excessive intake of fat. Wilcoxon matched-pairs signed rank test and McNemar paired Chi-square test were used to compare the differences in nutrient intake and the changes in the proportion of preschool children with excessive nutrient intake before and after modeling, respectively.

^a^Model 1 vs. Before simulation *P* < 0.05.

^b^Model 2 vs. Before simulation *P* < 0.05.

^c^Model 3 vs. Before simulation *P* < 0.05.

^d^Model 2 vs. Model 1 *P* < 0.05.

^e^Model 3 vs. Model 1 *P* < 0.05.

^f^Model 3 vs. Model 2 *P* < 0.05.

^g^Model 4 vs. Before simulation *P* < 0.05.

^h^Model 5 vs. Before simulation *P* < 0.05.

^i^Model 5 vs. Model 4 *P* < 0.05.

Model 1: The intake of liquid milk equivalents was replaced by soymilk at a matching volume.

Model 2: The intake of liquid milk equivalents was replaced by cow’s milk at a matching volume.

Model 3: The intake of liquid milk equivalents was replaced by FMP-PSC at a matching volume.

Model 4: The amount of cow’s milk was added to make the dairy intake of each child reach the recommended amount.

Model 5: The amount of FMP-PSC was added to make the dairy intake of each child reach the recommended amount.

### Mineral intakes after simulation

In scenario 1, although the calcium intake of children was significantly decreased following the substitution of soymilk (model 1), it was significantly increased after the simulation with cow’s milk (model 2), or FMP-PSC (model 3). Especially in the latter, the proportion of children with inadequate calcium intake decreased from 91.57 to 83.14%. Children’s iron and zinc intake changed similarly following the simulation, and only the replacement of FMP-PSC increased their intake. In addition, no improvement in inadequate iodine and potassium intake was seen as compared to the reported intake (Table 5).

**TABLE 5:** Mineral intakes after simulation (*n* = 676).

Minerals	Groups		*P*_50_ (*P*_25_, *P*_75_)	*N* (%)
Calcium (mg/d)	Before simulation		311.82 (214.98, 425.66)	619 (91.57%)
	Scenario 1	Model 1	148.30 (100.34, 216.65)^a^	674 (99.70%)^a^
		Model 2	324.63 (220.71, 451.34)^bd^	613 (90.68%)^d^
		Model 3	366.49 (242.29, 519.44)^cef^	562 (83.14%)^cef^
	Scenario 2	Model 4	502.06 (450.54, 574.62)^g^	510 (75.44%)^g^
		Model 5	559.27 (506.83, 622.25)^hi^	435 (64.35%)^hi^
Iron (mg/d)	Before simulation		9.19 (6.97, 12.02)	143 (21.15%)
	Scenario 1	Model 1	9.13 (6.82, 11.82)	152 (22.49%)
		Model 2	8.91 (6.67, 11.55)^bd^	164 (24.26%)^bd^
		Model 3	10.63 (8.13, 13.73)^cef^	94 (13.91%)^cef^
	Scenario 2	Model 4	9.69 (7.46, 12.41)^g^	107 (15.83%)^g^
		Model 5	11.42 (9.27, 14.11)^hi^	17 (2.51%)^hi^
Zinc (mg/d)	Before simulation		4.80 (3.59, 6.36)	254 (37.57%)
	Scenario 1	Model 1	4.38 (3.34, 5.70)^a^	295 (43.64%)^a^
		Model 2	4.66 (3.52, 6.02)^bd^	260 (38.46%)^d^
		Model 3	5.97 (4.41, 7.81)^cef^	149 (22.04%)^cef^
	Scenario 2	Model 4	5.51 (4.49, 6.94)^g^	124 (18.34%)^g^
		Model 5	6.96 (6.02, 8.24)^hi^	15 (2.22%)^hi^
Iodine (μg/d)	Before simulation		43.22 (16.10, 590.62)	394 (58.28%)
	Scenario 1	Model 1	35.95 (14.29, 592.21)^a^	400 (59.17%)
		Model 2	35.43 (13.75, 591.95)^bd^	401 (59.32%)
		Model 3	44.78 (22.29, 597.77)^cef^	383 (56.66%)^cef^
	Scenario 2	Model 4	46.58 (20.28, 593.94)^g^	389 (57.54%)
		Model 5	52.71 (30.01, 604.03)^hi^	371 (54.88%)^hi^
Potassium (mg/d)	Before simulation		824.26 (606.15, 1,134.74)	500 (73.96%)
	Scenario 1	Model 1	840.84 (630.50, 1,129.34)^a^	474 (70.12%)^a^
		Model 2	828.99 (622.01, 1,106.02)^d^	488 (72.19%)^d^
		Model 3	772.71 (571.04, 1,030.04)^cef^	526 (77.81%)^cef^
	Scenario 2	Model 4	1,031.31 (831.35, 1,295.77)^g^	379 (56.07%)^g^
		Model 5	962.59 (753.87, 1,225.69)^hi^	422 (62.43%)^hi^

The intakes of calcium, iron, zinc, and iodine below the estimated average requirement were perceived as inadequate, while the adequate intake was used for evaluating potassium inadequacy. Wilcoxon matched-pairs signed rank test and McNemar paired Chi-square test were used to compare the differences in nutrient intake and the changes in the proportion of preschool children with inadequate nutrient intake before and after modeling, respectively.

^a^Model 1 vs. Before simulation *P* < 0.05.

^b^Model 2 vs. Before simulation *P* < 0.05.

^c^Model 3 vs. Before simulation *P* < 0.05.

^d^Model 2 vs. Model 1 *P* < 0.05.

^e^Model 3 vs. Model 1 *P* < 0.05.

^f^Model 3 vs. Model 2 *P* < 0.05.

^g^Model 4 vs. Before simulation *P* < 0.05.

^h^Model 5 vs. Before simulation *P* < 0.05.

^i^Model 5 vs. Model 4 *P* < 0.05.

Model 1: The intake of liquid milk equivalents was replaced by soymilk at a matching volume.

Model 2: The intake of liquid milk equivalents was replaced by cow’s milk at a matching volume.

Model 3: The intake of liquid milk equivalents was replaced by FMP-PSC at a matching volume.

Model 4: The amount of cow’s milk was added to make the dairy intake of each child reach the recommended amount.

Model 5: The amount of FMP-PSC was added to make the dairy intake of each child reach the recommended amount.

In scenario 2, adding cow’s milk (model 4) or FMP-PSC milk (model 5) significantly increased calcium intake, and the proportion of children with inadequate calcium intake in the total population decreased by 16.13 and 27.22%, respectively. The addition of FMP-PSC significantly increased iron and zinc intake while decreasing the proportion of children with inadequate intake, which was 18.64 and 35.35%, respectively. Similar effects were also found in the addition of cow’s milk, but there was less improvement. The addition of cow’s milk and FMP-PSC both significantly increased children’s intake of iodine and potassium while tend to decrease the proportion of children with inadequate intake (Table 5).

The intakes of calcium, iron, zinc, iodine, and potassium by different age groups after simulation were shown in Supplementary Table 4 and the changes in these nutrients were similar to those of the whole age group.

### Vitamin intakes after simulation

In scenario 1, only the replacement of FMP-PSC (model 3) increased the intakes of vitamin A, vitamin B_1_, vitamin B_12_, vitamin C and vitamin D, while the opposite results were observed following the replacement of soymilk (model 1) or cow’s milk (model 2). The intakes of vitamin B_2_ and vitamin B_6_ were decreased following soymilk substitution, while their intakes were slightly increased after simulation with cow’s milk or FMP-PSC. In the models of soymilk and cow’s milk, the improvement of inadequate vitamin B_3_ intake was limited, but it was improved in the FMP-PSC model. After the simulation, Children’s vitamin B_9_ intake improved significantly, especially when soymilk was replaced. In the dairy food simulation, FMP-PSC improved on inadequate vitamin B_9_ intake better than cow’s milk, and the proportion of the total population with inadequate vitamin B_9_ intake decreased by 1.63 and 13.32%, respectively (Table 6).

**TABLE 6:** Vitamin intakes after simulation (*n* = 676).

Vitamins	Groups		*P*_50_ (*P*_25_, *P*_75_)	*N* (%)
Vitamin A (μg RAE/d)	Before simulation		211.57 (148.37, 293.54)	429 (63.46%)
	Scenario 1	Model 1	180.04 (123.94, 250.17)^a^	495 (73.22%)^a^
		Model 2	197.40 (136.50, 269.02)^bd^	451 (66.72%)^bd^
		Model 3	224.62 (160.35, 314.27)^cef^	371 (54.88%)^cef^
	Scenario 2	Model 4	251.52 (202.28, 326.33)^g^	321 (47.49%)^g^
		Model 5	286.74 (235.73, 354.90)^hi^	202 (29.88%)^hi^
Vitamin B_1_ (mg/d)	Before simulation		0.33 (0.24, 0.46)	596 (88.17%)
	Scenario 1	Model 1	0.31 (0.23, 0.42)^a^	621 (91.86%)^a^
		Model 2	0.33 (0.24, 0.44)^bd^	611 (90.38%)^bd^
		Model 3	0.37 (0.27, 0.49)^cef^	573 (84.76%)^cef^
	Scenario 2	Model 4	0.38 (0.31, 0.51)^g^	566 (83.73%)^g^
		Model 5	0.43 (0.35, 0.54)^hi^	538 (79.59%)^hi^
Vitamin B_2_ (mg/d)	Before simulation		0.53 (0.36, 0.73)	367 (54.29%)
	Scenario 1	Model 1	0.38 (0.28, 0.50)^a^	563 (83.28%)^a^
		Model 2	0.59 (0.43, 0.80)^bd^	315 (46.60%)^bd^
		Model 3	0.54 (0.40, 0.73)^cef^	359 (53.11%)^ef^
	Scenario 2	Model 4	0.78 (0.67, 0.94)^g^	62 (9.17%)^g^
		Model 5	0.74 (0.63, 0.90)^hi^	106 (15.68%)^hi^
Vitamin B_3_ (mg NE/d)	Before simulation		6.78 (5.12, 9.45)	249 (36.83%)
	Scenario 1	Model 1	6.89 (5.17, 9.51)^a^	244 (36.09%)
		Model 2	6.80 (5.08, 9.42)^bd^	251 (37.13%)^d^
		Model 3	7.31 (5.58, 10.05)^cef^	212 (31.36%)^cef^
	Scenario 2	Model 4	6.99 (5.24, 9.66)^g^	229 (33.88%)^g^
		Model 5	7.52 (5.70, 10.16)^hi^	188 (27.81%)^hi^
Vitamin B_6_ (mg/d)	Before simulation		0.71 (0.50, 0.93)	213 (31.51%)
	Scenario 1	Model 1	0.69 (0.49, 0.91)^a^	237 (35.06%)^a^
		Model 2	0.72 (0.52, 0.94)^bd^	213 (31.51%)^d^
		Model 3	0.72 (0.53, 0.95)^cef^	202 (29.88%)^cef^
	Scenario 2	Model 4	0.77 (0.58, 1.00)^g^	160 (23.67%)^g^
		Model 5	0.77 (0.59, 1.01)^hi^	150 (22.19%)^hi^
Vitamin B_9_ (μg DFE/d)	Before simulation		115.94 (84.86, 156.99)	465 (68.79%)
	Scenario 1	Model 1	182.27 (132.55, 242.33)^a^	202 (29.88%)^a^
		Model 2	118.08 (86.40, 161.04)^bd^	454 (67.16%)^bd^
		Model 3	137.73 (102.85, 181.64)^cef^	375 (55.47%)^cef^
	Scenario 2	Model 4	125.21 (94.79, 167.29)^g^	437 (64.64%)^g^
		Model 5	144.45 (113.62, 187.05)^hi^	333 (49.26%)^hi^
Vitamin B_12_ (μg/d)	Before simulation		1.63 (1.04, 2.54)	140 (20.71%)
	Scenario 1	Model 1	1.29 (0.88, 2.13)^a^	187 (27.66%)^a^
		Model 2	1.59 (1.03, 2.56)^d^	124 (18.34%)^bd^
		Model 3	1.89 (1.25, 2.93)^cef^	85 (12.57%)^cef^
	Scenario 2	Model 4	1.96 (1.49, 2.74)^g^	22 (3.25%)^g^
		Model 5	2.38 (1.96, 3.10)^hi^	2 (0.30%)^hi^
Vitamin C (mg/d)	Before simulation		42.47 (22.56, 69.87)	302 (44.67%)
	Scenario 1	Model 1	36.65 (19.42, 65.67)^a^	344 (50.89%)^a^
		Model 2	38.51 (20.53, 67.65)^bd^	333 (49.26%)^bd^
		Model 3	44.22 (26.14, 73.91)^cef^	289 (42.75%)^cef^
	Scenario 2	Model 4	44.24 (24.40, 72.04)^g^	297 (43.93%)
		Model 5	49.64 (30.63, 77.03)^hi^	255 (37.72%)^hi^
Vitamin D (μg/d)	Before simulation		0.00 (0.00, 1.82)	666 (98.52%)
	Scenario 1	Model 1	0.00 (0.00, 0.00)^a^	670 (99.11%)
		Model 2	0.00 (0.00, 0.00)^b^	670 (99.11%)
		Model 3	1.25 (0.61, 1.96)^cef^	667 (98.67%)
	Scenario 2	Model 4	0.00 (0.00, 1.82)^g^	666 (98.52%)
		Model 5	2.12 (1.42, 2.86)^hi^	665 (98.37%)

RAE, retinol activity equivalent; NE, nicotinic acid equivalent; DFE, dietary folate equivalent. The intakes of vitamins below the estimated average requirement were perceived as inadequate. Wilcoxon matched-pairs signed rank test and McNemar paired Chi-square test were used to compare the differences in nutrient intake and the changes in the proportion of preschool children with inadequate nutrient intake before and after modeling, respectively.

^a^Model 1 vs. Before simulation *P* < 0.05.

^b^Model 2 vs. Before simulation *P* < 0.05.

^c^Model 3 vs. Before simulation *P* < 0.05.

^d^Model 2 vs. Model 1 *P* < 0.05.

^e^Model 3 vs. Model 1 *P* < 0.05.

^f^Model 3 vs. Model 2 *P* < 0.05.

^g^Model 4 vs. Before simulation *P* < 0.05.

^h^Model 5 vs. Before simulation *P* < 0.05.

^i^Model 5 vs. Model 4 *P* < 0.05.

Model 1: The intake of liquid milk equivalents was replaced by soymilk at a matching volume.

Model 2: The intake of liquid milk equivalents was replaced by cow’s milk at a matching volume.

Model 3: The intake of liquid milk equivalents was replaced by FMP-PSC at a matching volume.

Model 4: The amount of cow’s milk was added to make the dairy intake of each child reach the recommended amount.

Model 5: The amount of FMP-PSC was added to make the dairy intake of each child reach the recommended amount.

In scenario 2, increasing FMP-PSC (model 5) significantly raised the intakes of vitamin A, vitamin B_1_, vitamin B_12_, and vitamin C, and decreased the proportion of children with inadequate intakes, which were 33.58, 8.58, 20.41, and 6.95%, respectively. Although the addition of cow’s milk had comparable effects (model 4), there was less improvement. The inadequate intake of vitamin B_2_ and vitamin B_6_ was significantly improved, and the effect of adding cow’s milk was greater than that of adding FMP-PSC. The improvement of inadequate intake of vitamin B_3_ and vitamin D was limited in the model of cow’s milk, but this situation improved in the FMP-PSC model. Furthermore, FMP-PSC improved on inadequate vitamin B_9_ intake better than cow’s milk, reducing the proportion of children with inadequate vitamin B_9_ intake by 19.53% (Table 6).

The intakes of vitamin A, vitamin B_1_, vitamin B_2_, vitamin B_3_, vitamin B_6_, vitamin B_9_, vitamin B_12_, vitamin C, and vitamin D by different age groups after simulation were shown in Supplementary Table 4 and the changes in these nutrients were similar to those of the whole age group.

## Discussion

The DSIYC, 2018–2019, on which this simulation study was based, is a cross-sectional survey of Chinese children aged 0–6 years conducted in multiple regions of China from 2018 to 2019. It can be seen from the results that the percentage of consumption and amounts of dairy products consumed have increased in recent years, but the consumption of dairy products by preschool children remains low. According to CHNS 2015, 97.6% of children did not meet the recommended 300 g/day of dairy foods (16). We found that 88.31% of preschool children in this study did not meet the recommended amount (350 g/day). As a result, more research into the impact of dairy products on the dietary nutritional status of preschool children is required.

This study also looked at the trends in dairy consumption among age groups. The results showed that the intake of total dairy products decreased significantly with age, which was consistent with the findings of other studies in China and other countries (23, 24). However, the results of this study’s change in dairy types with age differed from those of other countries. In western countries, for example, a study in Germany found that with the increase of age (3.5–18.5 years), the type of dairy products changed from liquid to solid, and the intake of fermented dairy products increased (24). However, in this study, liquid milk was the most consumed dairy item, followed by milk powder, while the consumption of fermented dairy products (such as yogurt and cheese) was lower. Only the proportion of children consuming milk powder, not liquid milk or fermented dairy products, declined considerably with age. It is worth mentioning that the formula milk powder is 86.64% of the milk powder consumed by children aged 3–6 in this study. As a result, formula milk powder played a role in improving preschool children’s dietary nutrition, and this study compared the effects of liquid milk and FMP-PSC on children’s dietary nutrition by simulating two scenarios. Soymilk, unlike popular milk in western countries, is a food with Chinese characteristics that is widely popular in China (25). Therefore, in scenario 1, we included a simulation that replaced soymilk for all dairy products in the reported data to compare the effects of soymilk, cow’s milk, and FMP-PSC on children’s dietary nutrition.

Previous studies have shown that dairy products aid in the adequate intake of nutrients for children and adolescents. According to the data from the 2007 Australian National Children’s Nutrition and Physical Activity Survey (2–16 years), consuming milk was associated with increased calcium, phosphorus, magnesium, potassium, and iodine intakes when compared to those who did not drink milk (26). According to National Health and Nutrition Examination Survey 2001–2016 data, American children who drank yogurt took in more calcium, magnesium, potassium, sodium, vitamin B_12_, and vitamin D than non-consumers (27). Data from the South East Asian Nutrition Survey (0.5–12 years) showed that dairy as part of a daily diet supported a healthy vitamin A and vitamin D status (28). Our study yielded comparable findings. In scenario 1, only the intake of dietary fiber, potassium, and vitamin B_9_ was significantly increased after replacing all dairy products in the reported data with soymilk. While the intake of calcium, vitamin B_2_, vitamin B_3_, vitamin B_6_, and vitamin B_9_ was significantly higher after replacing all dairy products in the reported data with cow’s milk or FMP-PSC; and the impact of FMP-PSC was significantly better than that of cow’s milk in increasing the intake of calcium, vitamin B_3_, vitamin B_6_, and vitamin B_9_. Other simulation studies on children’s dairy products had similar results (16, 29). Interestingly, our study found that replacing all dairy products in the reported data with cow’s milk considerably reduced children’s intake of dietary fiber, DHA, iron, zinc, iodine, vitamin A, vitamin B_1_, vitamin B_12_, vitamin C, and vitamin D. However, when all dairy products in the reported data were replaced with FMP-PSC, the intake of these nutrients rose significantly. This situation may be explained by the reported data’s high proportion of formula milk powder consumed by preschool children. More and more Chinese families take FMP-PSC as a part of children’s daily diet to meet their nutritional needs for growth and development. The composition of cow’s milk and FMP-PSC is different. On top of the nutritional content of cow’s milk, including high quality protein and high calcium content, FMP-PSC is further fortified with several micronutrients and functional ingredients, which play an important role in preventing children from nutrient deficiency and maintaining their health (30, 31).

The adequacy of children’s dairy intake is positively correlated with higher nutritional intake, nutritional adequacy, and dietary quality. Dairy products are important for bone health and linear growth in childhood. A previous study indicated that promoting dairy consumption may be a feasible and effective measure to improve the linear growth of Chinese preschool children (32). In addition, many studies support the inverse association between the amount of dairy intake and the indicators of obesity, dental caries, and hypertension in children and adolescents (33–35). Therefore, this study simulated dairy products achieving the recommended amount after adding liquid milk or FMP-PSC. The results showed that adding cow’s milk or FMP-PSC significantly increased the intake levels of protein and some key micronutrients, including calcium, iron, zinc, iodine, potassium, vitamin A, vitamin B_1_, vitamin B_2_, vitamin B_3_, vitamin B_6_, vitamin B_9_, vitamin B_12_, and vitamin C. Among these micronutrients, except for potassium and vitamin B_2_, the improvement effect of FMP-PSC on inadequate intakes of micronutrients was significantly better than that of cow’s milk. This result is similar to the previous simulation study based on the dietary survey data of children (1–8 years) in China (16). In addition, due to the lack of dietary fiber and DHA in cow’s milk, the addition of cow’s milk did not significantly increase the intake of these two nutrients as the addition of FMP-PSC, which is also the advantage of FMP-PSC over cow’s milk.However, based on the results of this study, we must admit that there are some limitations. First, though food fortification, such as FMP-PSC is a good way to improve vitamin D intake, the study shows the prevalence of insufficient vitamin D intake remains high. Since vitamin D status is closely linked to lifestyle, in future research, lifestyle factors such as outdoor activity time and sunscreen application should be considered to better assess vitamin D status. Moreover, the simulation scenarios of this study only used liquid milk equivalent to determine the dietary nutritional status of various models. In fact, the consumption patterns of dairy products are diversified, and a larger sample size will be required to evaluate more complex simulation scenarios, such as the combination of different types of dairy products (liquid milk, yogurt, cheese, condensed milk, etc.) and FMP-YCF.

In conclusion, consuming dairy products below the recommended level leads to inadequate nutrient intake in preschool children. Since food preferences and dietary behaviors develop in the preschool period, determining the most effective dietary behaviors to prevent the decline of dairy intake in preschool children is critical. Our simulation study showed that replacing dairy foods with FMP-PSC at matching volume (scenario 1) is better than replacing with soymilk or cow’s milk in increasing most nutrient intakes in preschool children and adding FMP-PSC to make the intake of dairy products per child reach the recommended amount (scenario 2) can bring the intake of most nutrients of preschool children more in line with the recommendations when compared to cow’s milk. Therefore, to guide the scientific consumption of dairy products, correct nutrition information should be given to parents and preschool children. When necessary, the use and addition of dairy products with food ingredients or nutritional fortifiers can be encouraged. Furthermore, for optimal nutritional intakes of preschool children, in addition to the dietary behavior modification of dairy consumption, a multi-faceted approach that includes a diversified balanced diet and the use of dietary supplements (such as vitamin D) is required.

## Data availability statement

The original contributions presented in this study are included in the article/Supplementary material, further inquiries can be directed to the corresponding author.

## Author contributions

ZW and YD: conceptualization and design of the work. YD, ZX, GL, YZ, JY, and MF: analysis and interpretation of the work. YD, FH, and GL: writing—original draft preparation. YD, ZX, FH, JY, and ZW: writing—review and editing. ZW: supervision and funding acquisition. All authors contributed to the article and approved the submitted version.
