# Supplementary material for: Dairy fortification as a good option for dietary nutrition status improvement of 676 preschool children in China: A simulation study based on a cross-sectional diet survey (2018–2019)
Source: Front Nutr. 2022 Dec 8;9:1081495. doi: 10.3389/fnut.2022.1081495 (PMC9773072; doi:10.3389/fnut.2022.1081495)
Supplement: Supplementary file 1 [file Data_Sheet_1.docx]

Supplementary Material

# Supplementary Figure and Tables

## Supplementary Figure


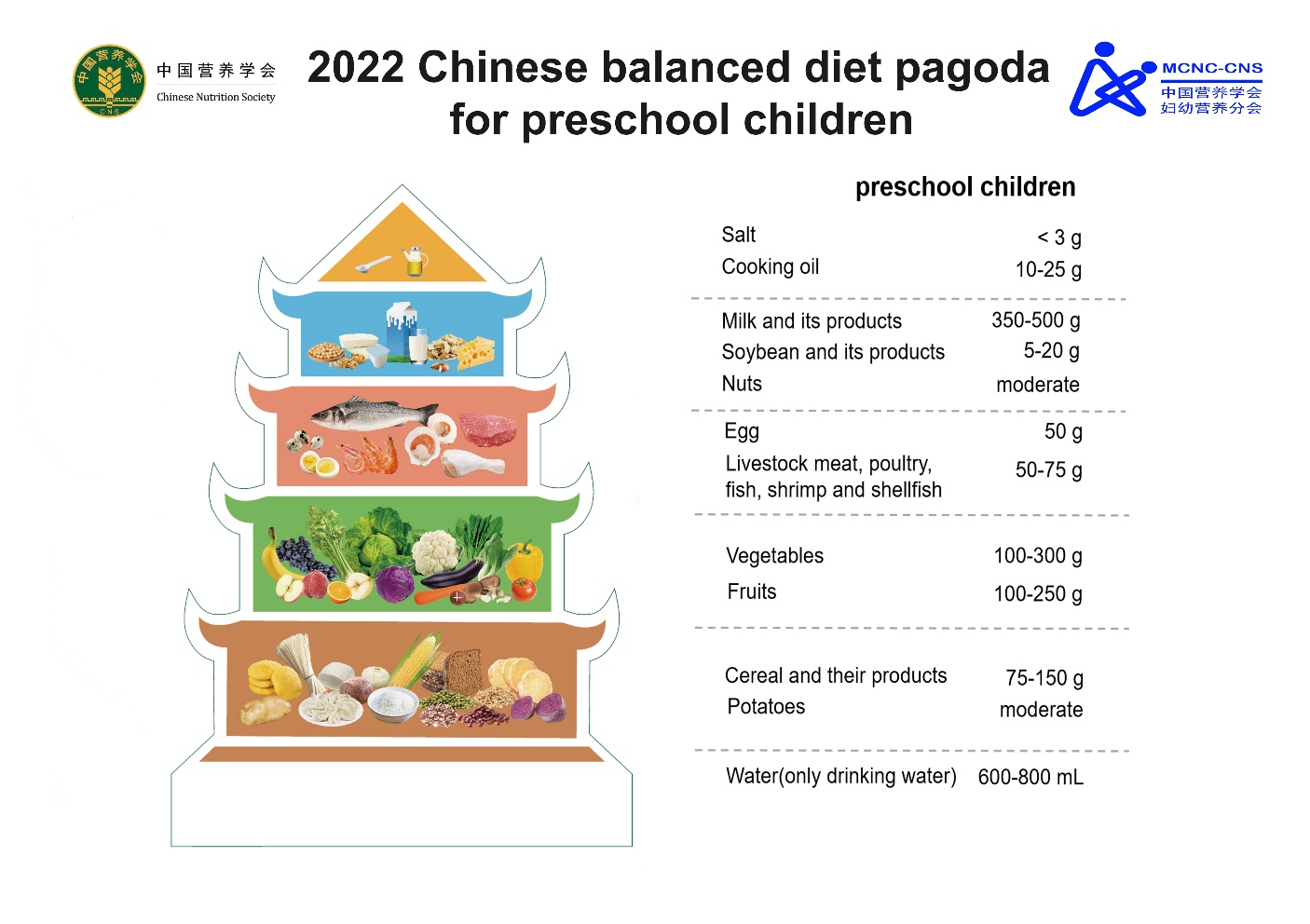


**Supplementary Figure 1.** 2022 Chinese balanced diet pagoda for preschool children.

## Supplementary Tables

**Supplementary Table 1.** Nutritional composition per 100 g of soymilk, cow’s milk, and FMP-PSC used for simulation

| Parameters | Soymilk | Cow’s milk | FMP-PSC |
| --- | --- | --- | --- |
| Energy (kcal) | 31 | 54 | 456.8 |
| Carbohydrate (g) | 1.2 | 3.4 | 44.7 |
| Protein (g) | 3 | 3 | 19.4 |
| Fat (g) | 1.6 | 3.2 | 20.7 |
| Dietary fiber (g) | 1.1 | / | 7 |
| DHA (mg) | / | / | 54 |
| Calcium (mg) | 5 | 104 | 867 |
| Iron (mg) | 0.4 | 0.3 | 8.2 |
| Zinc (mg) | 0.28 | 0.42 | 7.74 |
| Iodine (µg) | 2.1 | 1.9 | 41.9 |
| Potassium (mg) | 117 | 109 | 462 |
| Vitamin A (μg RE) | 15 | 24 | 276 |
| Vitamin B_1_ (mg) | 0.02 | 0.03 | 0.35 |
| Vitamin B_2_ (mg) | 0.02 | 0.14 | 0.77 |
| Vitamin B_3_ (mg) | 0.14 | 0.1 | 2.48 |
| Vitamin B_6_ (mg) | 0.019 | 0.036 | 0.27 |
| Vitamin B_9_ (μg DFE) | 39.4 | 5 | 100 |
| Vitamin B_12_ (μg) | 0.1 | 0.2 | 2.7 |
| Vitamin C (mg) | / | 1 | 26.3 |
| Vitamin D (μg) | / | / | 4.7 |

FMP-PSC: formulated milk powder for preschool children (Aptamil); RE: retinol equivalent; DFE: dietary folate equivalent.

**Supplementary Table 2.** Macronutrient intakes by different age groups after simulation

| **Macronutrients** | **Groups** | | **37~48 months (n=224)** | |  | **49~60 months (n=226)** | |  | **61~72 months (n=226)** | |
| --- | --- | --- | --- | --- | --- | --- | --- | --- | --- | --- |
|  |  |  | ***P*_50_ (*P*_25_, *P*_75_)** | **%** |  | ***P*_50_ (*P*_25_, *P*_75_)** | **%** |  | ***P*_50_ (*P*_25_, *P*_75_)** | **%** |
| **Carbohydrate**  **(g/d)** | Before simulation | | 104.00 (77.79, 141.21) | 62.95 |  | 108.84 (85.63, 137.35) | 59.73 |  | 120.16 (87.58, 157.03) | 49.56 |
|  | Scenario 1 | Model 1 | 93.27 (64.65, 128.52) ^a^ | 69.64 ^a^ |  | 98.75 (78.43, 124.73) ^a^ | 69.91 ^a^ |  | 109.75 (77.92, 149.78) ^a^ | 57.52 ^a^ |
|  |  | Model 2 | 97.57 (69.37, 133.58) ^bd^ | 68.75 ^b^ |  | 102.90 (81.76, 128.90) ^bd^ | 64.16 ^bd^ |  | 114.29 (84.99, 150.46) ^bd^ | 54.42 ^bd^ |
|  |  | Model 3 | 104.03 (76.67, 139.86) ^cef^ | 63.39 ^ef^ |  | 108.75 (86.13, 136.57) ^ef^ | 60.18 ^ef^ |  | 118.08 (87.60, 155.78) ^e^ | 50.44 ^ef^ |
|  | Scenario 2 | Model 4 | 111.46 (81.32, 146.22) ^g^ | 57.59 ^g^ |  | 114.78 (92.50, 141.28) ^g^ | 55.75 ^g^ |  | 127.63 (96.72, 162.71) ^g^ | 46.02 ^g^ |
|  |  | Model 5 | 118.14 (86.10, 151.81) ^hi^ | 54.02 ^hi^ |  | 120.58 (97.63, 146.31) ^hi^ | 50.00 ^hi^ |  | 132.29 (103.89, 170.27) ^hi^ | 42.48 ^hi^ |
| **Protein**  **(g/d)** | Before simulation | | 31.37 (23.75, 41.26) | 27.68 |  | 32.59 (25.64, 44.50) | 22.57 |  | 36.46 (26.78, 43.36) | 21.24 |
|  | Scenario 1 | Model 1 | 31.86 (24.23, 42.14) ^a^ | 26.79 |  | 32.77 (25.84, 45.26) ^a^ | 22.57 |  | 36.57 (26.63, 43.48) ^a^ | 21.24 |
|  |  | Model 2 | 31.86 (24.23, 42.14) ^b^ | 26.79 |  | 32.77 (25.84, 45.26) ^b^ | 22.57 |  | 36.56 (26.83, 43.48) ^b^ | 21.24 |
|  |  | Model 3 | 31.69 (24.13, 41.97) ^cef^ | 27.23 |  | 32.67 (25.71, 45.07) ^cef^ | 22.57 |  | 36.51 (26.56, 43.17) ^e^ | 21.68 |
|  | Scenario 2 | Model 4 | 35.84 (29.48, 45.46) ^g^ | 12.50 ^g^ |  | 37.52 (31.37, 46.91) ^g^ | 9.29 ^g^ |  | 41.57 (34.45, 49.00) ^g^ | 7.08 ^g^ |
|  |  | Model 5 | 35.75 (29.41, 45.34) ^hi^ | 12.50 ^h^ |  | 37.40 (31.24, 46.84) ^hi^ | 0.29 ^h^ |  | 41.47 (34.38, 48.83) ^hi^ | 7.52 ^h^ |
| **Fat**  **(g/d)** | Before simulation | | 34.76 (27.97, 42.55) | / |  | 39.50 (31.75, 50.84) | / |  | 40.01 (31.19, 52.42) | / |
|  | Scenario 1 | Model 1 | 29.87 (24.36, 38.11) ^a^ | / |  | 35.68 (28.32, 44.83) ^a^ | / |  | 37.15 (29.31, 47.37) ^a^ | / |
|  |  | Model 2 | 33.52 (26.76, 41.93) ^bd^ | / |  | 38.91 (30.55, 50.24) ^bd^ | / |  | 39.60 (30.71, 51.49) ^bd^ | / |
|  |  | Model 3 | 33.38 (26.74, 41.82) ^cef^ | / |  | 38.76 (30.37, 50.11) ^cef^ | / |  | 39.47 (30.76, 51.09) ^cef^ | / |
|  | Scenario 2 | Model 4 | 39.42 (33.94, 47.59) ^g^ | / |  | 44.90 (38.19, 53.82) ^g^ | / |  | 46.71 (38.88, 56.13) ^g^ | / |
|  |  | Model 5 | 39.28 (33.70, 47.44) ^hi^ | / |  | 44.75 (38.03, 53.64) ^hi^ | / |  | 46.53 (38.73, 56.07) ^hi^ | / |
| **Dietary fiber**  **(g/d)** | Before simulation | | 3.95 (2.68, 5.75) | 99.11 |  | 3.76 (2.64, 5.70) | 99.56 |  | 4.17 (3.00, 6.25) | 97.79 |
|  | Scenario 1 | Model 1 | 6.14 (4.43, 8.21) ^a^ | 78.13 ^a^ |  | 5.74 (4.38, 8.32) ^a^ | 92.92 ^a^ |  | 6.43 (4.03, 8.41) ^a^ | 86.73 ^a^ |
|  |  | Model 2 | 3.95 (2.68, 5.75) ^d^ | 98.21 ^d^ |  | 3.76 (2.64, 5.70) ^d^ | 99.56 ^d^ |  | 4.17 (3.00, 6.25) ^bd^ | 96.90 ^d^ |
|  |  | Model 3 | 6.07 (4.40, 8.09) ^cef^ | 92.41 ^cef^ |  | 5.66 (4.32, 8.16) ^cef^ | 96.90 ^cef^ |  | 6.26 (4.00, 8.27) ^cef^ | 92.92 ^cef^ |
|  | Scenario 2 | Model 4 | 3.95 (2.68, 5.75) | 100.00 |  | 3.76 (2.64, 5.70) ^g^ | 99.56 |  | 4.17 (3.00, 6.25) ^g^ | 98.23 |
|  |  | Model 5 | 6.02 (4.48, 7.68) ^hi^ | 97.32 ^i^ |  | 5.82 (4.67, 7.49) ^hi^ | 99.12 |  | 6.61 (5.10, 8.23) ^hi^ | 95.58 ^i^ |
| **DHA**  **(mg/d)** | Before simulation | | 15.65 (9.10, 26.25) | / |  | 15.86 (8.98, 25.83) | / |  | 16.79 (8.73, 26.74) | / |
|  | Scenario 1 | Model 1 | 11.32 (6.80, 19.55) ^a^ | / |  | 13.16 (7.96, 23.55) ^a^ | / |  | 14.73 (7.48, 25.57) ^a^ | / |
|  |  | Model 2 | 11.32 (6.83, 19.55) ^b^ | / |  | 13.16 (7.96, 23.55) ^b^ | / |  | 14.82 (7.42, 25.66) ^b^ | / |
|  |  | Model 3 | 28.03 (17.01, 42.59) ^cef^ | / |  | 28.39 (17.84, 43.40) ^cef^ | / |  | 28.77 (16.87, 42.58) ^cef^ | / |
|  | Scenario 2 | Model 4 | 15.65 (9.10, 26.25) | / |  | 15.86 (8.98, 25.83) ^g^ | / |  | 16.79 (8.73, 26.74) | / |
|  |  | Model 5 | 30.42 (24.22, 39.40) ^hi^ | / |  | 32.35 (25.64, 40.01) ^hi^ | / |  | 33.64 (26.24, 42.92) ^hi^ | / |

The intakes of carbohydrate and protein below the estimated average requirement were perceived as inadequate, while inadequate fiber intake was defined as the daily intake of less than 10g/1000 kcal energy.

Wilcoxon matched-pairs signed rank test and McNemar paired Chi-square test were used to compare the differences in nutrient intake and the changes in the proportion of preschool children with inadequate nutrient intake before and after modeling, respectively.

^a^: Model 1 *vs.* Before simulation *P* < 0.05; ^b^: Model 2 *vs.* Before simulation *P* < 0.05; ^c^: Model 3 *vs.* Before simulation *P* < 0.05; ^d^: Model 2 *vs.* Model 1 *P* < 0.05; ^e^: Model 3 *vs.* Model 1 *P* < 0.05; ^f^: Model 3 *vs.* Model 2 *P* < 0.05; ^g^: Model 4 *vs.* Before simulation *P* < 0.05; ^h^: Model 5 *vs.* Before simulation *P* < 0.05; ^i^: Model 5 *vs.* Model 4 *P* < 0.05.

Model 1: The intake of liquid milk equivalents was replaced by soymilk at a matching volume.

Model 2: The intake of liquid milk equivalents was replaced by cow’s milk at a matching volume.

Model 3: The intake of liquid milk equivalents was replaced by FMP-PSC at a matching volume.

Model 4: The amount of cow’s milk was added to make the dairy intake of each child reach the recommended amount.

Model 5: The amount of FMP-PSC was added to make the dairy intake of each child reach the recommended amount.

**Supplementary Table 3.** Mineral intakes by different age groups after simulation

| **Minerals** | **Groups** | | **37~48 months (n=224)** | |  | **49~60 months (n=226)** | |  | **61~72 months (n=226)** | |
| --- | --- | --- | --- | --- | --- | --- | --- | --- | --- | --- |
|  |  |  | ***P*_50_ (*P*_25_, *P*_75_)** | **%** |  | ***P*_50_ (*P*_25_, *P*_75_)** | **%** |  | ***P*_50_ (*P*_25_, *P*_75_)** | **%** |
| **Calcium**  **(mg/d)** | Before simulation | | 313.36 (228.27, 423.87) | 83.48 |  | 310.66 (222.45, 413.98) | 95.13 |  | 309.62 (205.01, 435.00) | 96.02 |
|  | Scenario 1 | Model 1 | 148.83 (97.75, 210.42) ^a^ | 99.55 ^a^ |  | 146.25 (101.32, 204.81) ^a^ | 100.00 ^a^ |  | 153.98 (100.97, 225.36) ^a^ | 99.56 ^a^ |
|  |  | Model 2 | 336.95 (231.90, 460.80) ^bd^ | 81.25 ^d^ |  | 325.27 (235.37, 449.08) ^bd^ | 92.92 ^d^ |  | 307.86 (204.38, 449.02) ^bd^ | 97.79 |
|  |  | Model 3 | 382.03 (255.00, 533.72) ^cef^ | 70.98 ^cef^ |  | 373.96 (255.36, 520.88) ^cef^ | 88.94 ^cef^ |  | 343.32 (228.24, 493.66) ^cef^ | 89.38 ^cef^ |
|  | Scenario 2 | Model 4 | 501.72 (445.49, 569.90) ^g^ | 49.11 ^g^ |  | 493.25 (441.77, 567.88) ^g^ | 87.61 ^g^ |  | 508.49 (460.56, 584.77) ^g^ | 89.38 ^g^ |
|  |  | Model 5 | 555.24 (489.95, 620.05) ^hi^ | 28.57 ^hi^ |  | 550.47 (498.42, 615.77) ^hi^ | 84.51 ^hi^ |  | 574.23 (524.83, 629.69) ^hi^ | 79.65 ^hi^ |
| **Iron**  **(mg/d)** | Before simulation | | 9.22 (6.73, 11.73) | 15.63 |  | 8.70 (6.97, 11.57) | 25.66 |  | 9.70 (7.19, 12.82) | 22.12 |
|  | Scenario 1 | Model 1 | 8.71 (6.53, 10.94) ^a^ | 17.41 |  | 8.82 (6.81, 11.25) | 28.76 |  | 9.62 (7.39, 12.93) ^a^ | 21.24 |
|  |  | Model 2 | 8.57 (6.37, 10.69) ^bd^ | 20.54 ^bd^ |  | 8.67 (6.62, 10.98) ^bd^ | 30.97 ^b^ |  | 9.51 (7.23, 12.70) ^bd^ | 21.24 |
|  |  | Model 3 | 10.54 (8.16, 13.28) ^cef^ | 10.71 ^cef^ |  | 10.21 (8.00, 13.56) ^cef^ | 15.49 ^cef^ |  | 11.15 (8.43, 14.40) ^cef^ | 15.49 ^cef^ |
|  | Scenario 2 | Model 4 | 9.68 (7.28, 12.13) ^g^ | 11.61 ^g^ |  | 9.26 (7.42, 11.93) ^g^ | 17.70 ^g^ |  | 10.27 (7.72, 13.27) ^g^ | 18.14 ^g^ |
|  |  | Model 5 | 11.24 (9.08, 13.74) ^hi^ | 1.79 ^hi^ |  | 11.11 (9.25, 13.22) ^hi^ | 1.77 ^hi^ |  | 12.24 (9.81, 14.82) ^hi^ | 3.98 ^hi^ |
| **Zinc**  **(mg/d)** | Before simulation | | 4.69 (3.56, 6.09) | 19.20 |  | 4.69 (3.53, 6.52) | 48.67 |  | 4.93 (3.61, 6.41) | 44.69 |
|  | Scenario 1 | Model 1 | 4.26 (3.24, 5.43) ^a^ | 24.55 ^a^ |  | 4.24 (3.28, 5.93) ^a^ | 57.52 ^a^ |  | 4.69 (3.47, 5.80) ^a^ | 48.67 ^a^ |
|  |  | Model 2 | 4.45 (3.45, 5.84) ^bd^ | 18.75 ^d^ |  | 4.51 (3.49, 6.23) ^bd^ | 51.77 ^d^ |  | 4.89 (3.65, 6.08) ^bd^ | 44.69 ^d^ |
|  |  | Model 3 | 5.93 (4.45, 7.54) ^cef^ | 8.93 ^cef^ |  | 5.98 (4.51, 7.97) ^cef^ | 26.11 ^cef^ |  | 5.99 (4.20, 8.04) ^cef^ | 30.97 ^cef^ |
|  | Scenario 2 | Model 4 | 5.39 (4.40, 6.77) ^g^ | 1.79 ^g^ |  | 5.37 (4.42, 7.04) ^g^ | 28.76 ^g^ |  | 5.78 (4.62, 6.97) ^g^ | 24.34 ^g^ |
|  |  | Model 5 | 6.69 (5.86, 8.15) ^hi^ | 0.00 ^h^ |  | 6.83 (5.94, 8.18) ^hi^ | 2.65 ^hi^ |  | 7.38 (6.28, 8.46) ^hi^ | 3.98 ^hi^ |
| **Iodine**  **(µg/d)** | Before simulation | | 47.72 (18.71, 602.03) | 57.14 |  | 35.53 (15.09, 553.08) | 62.39 |  | 49.48 (15.67, 581.65) | 55.31 |
|  | Scenario 1 | Model 1 | 31.39 (14.21, 595.15) ^a^ | 58.48 |  | 30.33 (13.91, 551.69) ^a^ | 62.39 |  | 51.09 (16.02, 587.90) ^a^ | 56.64 |
|  |  | Model 2 | 31.06 (13.72, 594.87) ^bd^ | 58.48 |  | 30.01 (13.55, 551.16) ^bd^ | 62.39 |  | 46.20 (15.17, 578.62) ^d^ | 57.08 |
|  |  | Model 3 | 40.94 (22.80, 604.85) ^cef^ | 55.80 ^ef^ |  | 40.76 (20.09, 555.20) ^cef^ | 60.62 |  | 54.26 (22.83, 582.89) ^cef^ | 53.54 ^ef^ |
|  | Scenario 2 | Model 4 | 48.45 (21.75, 604.77) ^g^ | 56.25 |  | 38.50 (18.55, 559.05) ^g^ | 61.06 |  | 51.30 (20.26, 585.76) ^g^ | 55.31 |
|  |  | Model 5 | 53.54 (30.28, 610.94) ^hi^ | 54.91 |  | 44.62 (29.25, 567.59) ^hi^ | 57.96 ^hi^ |  | 58.99 (31.89, 592.98) ^hi^ | 51.77 ^hi^ |
| **Potassium**  **(mg/d)** | Before simulation | | 824.29 (626.89, 1085.75) | 60.71 |  | 799.76 (582.37, 1135.44) | 83.63 |  | 871.97 (600.21, 1163.62) | 77.43 |
|  | Scenario 1 | Model 1 | 834.06 (644.86, 1100.91) | 54.46 ^a^ |  | 826.55 (616.99, 1124.59) | 78.76 ^a^ |  | 871.29 (633.03, 1168.45) ^a^ | 76.99 |
|  |  | Model 2 | 822.86 (629.21, 1096.61) ^d^ | 56.70 |  | 810.38 (604.12, 1107.56) ^d^ | 80.53 |  | 864.83 (630.12, 1138.37) ^d^ | 79.20 |
|  |  | Model 3 | 759.31 (566.49, 1018.51) ^cef^ | 67.41 ^cef^ |  | 756.63 (551.32, 999.80) ^cef^ | 83.63 ^ef^ |  | 790.34 (594.49, 1049.22) ^cef^ | 82.30 ^cef^ |
|  | Scenario 2 | Model 4 | 1014.23 (823.24, 1271.54) ^g^ | 36.16 ^g^ |  | 1023.31 (822.47, 1255.79) ^g^ | 69.91 ^g^ |  | 1077.90 (863.95, 1346.29) ^g^ | 61.95 ^g^ |
|  |  | Model 5 | 939.22 (751.88, 1196.23) ^hi^ | 45.09 ^hi^ |  | 941.00 (736.70, 1199.78) ^hi^ | 75.22 ^hi^ |  | 1002.08 (780.00, 1273.15) ^hi^ | 66.81 ^hi^ |

The intakes of calcium, iron, zinc and iodine below the estimated average requirement were perceived as inadequate, while adequate intake was used for evaluating potassium inadequacy.

Wilcoxon matched-pairs signed rank test and McNemar paired Chi-square test were used to compare the differences in nutrient intake and the changes in the proportion of preschool children with inadequate nutrient intake before and after modeling, respectively.

^a^: Model 1 *vs.* Before simulation *P* < 0.05; ^b^: Model 2 *vs.* Before simulation *P* < 0.05; ^c^: Model 3 *vs.* Before simulation *P* < 0.05; ^d^: Model 2 *vs.* Model 1 *P* < 0.05; ^e^: Model 3 *vs.* Model 1 *P* < 0.05; ^f^: Model 3 *vs.* Model 2 *P* < 0.05; ^g^: Model 4 *vs.* Before simulation *P* < 0.05; ^h^: Model 5 *vs.* Before simulation *P* < 0.05; ^i^: Model 5 *vs.* Model 4 *P* < 0.05.

Model 1: The intake of liquid milk equivalents was replaced by soymilk at a matching volume.

Model 2: The intake of liquid milk equivalents was replaced by cow’s milk at a matching volume.

Model 3: The intake of liquid milk equivalents was replaced by FMP-PSC at a matching volume.

Model 4: The amount of cow’s milk was added to make the dairy intake of each child reach the recommended amount.

Model 5: The amount of FMP-PSC was added to make the dairy intake of each child reach the recommended amount.

**Supplementary Table 4.** Vitamin intakes by different age groups after simulation

| **Vitamins** | **Groups** | | **37~48 months (n=224)** | |  | **49~60 months (n=226)** | |  | **61~72 months (n=226)** | |
| --- | --- | --- | --- | --- | --- | --- | --- | --- | --- | --- |
|  |  |  | ***P*_50_ (*P*_25_, *P*_75_)** | **%** |  | ***P*_50_ (*P*_25_, *P*_75_)** | **%** |  | ***P*_50_ (*P*_25_, *P*_75_)** | **%** |
| **Vitamin A**  **(μgRAE/d)** | Before simulation | | 203.98 (152.29, 293.22) | 54.46 |  | 218.55 (146.58, 290.25) | 66.37 |  | 207.95 (146.04, 294.09) | 69.47 |
|  | Scenario 1 | Model 1 | 178.59 (126.20, 244.57) ^a^ | 64.73 ^a^ |  | 180.60 (123.23, 251.84) ^a^ | 77.88 ^a^ |  | 181.73 (122.67, 253.37) ^a^ | 76.99 ^a^ |
|  |  | Model 2 | 198.31 (143.71, 261.88) ^bd^ | 58.04 ^bd^ |  | 195.87 (133.83, 274.94) ^bd^ | 70.35 ^bd^ |  | 197.40 (135.53, 267.29) ^bd^ | 71.68 ^d^ |
|  |  | Model 3 | 226.55 (168.80, 318.51) ^cef^ | 45.09 ^cef^ |  | 224.73 (160.88, 320.38) ^cef^ | 57.52 ^cef^ |  | 222.42 (148.90, 303.41) ^cef^ | 61.95 ^cef^ |
|  | Scenario 2 | Model 4 | 249.07 (198.80, 323.99) ^g^ | 37.95 ^g^ |  | 257.31 (201.96, 330.09) ^g^ | 51.77 ^g^ |  | 256.66 (206.43, 324.33) ^g^ | 52.65 ^g^ |
|  |  | Model 5 | 277.06 (230.74, 354.80) ^hi^ | 18.30 ^hi^ |  | 285.08 (236.39, 355.20) ^hi^ | 37.61 ^hi^ |  | 290.52 (241.66, 355.13) ^hi^ | 33.63 ^hi^ |
| **Vitamin B_1_**  **(mg/d)** | Before simulation | | 0.33 (0.25, 0.46) | 80.36 |  | 0.32 (0.24, 0.46) | 91.59 |  | 0.35 (0.23, 0.45) | 92.48 |
|  | Scenario 1 | Model 1 | 0.29 (0.22, 0.41) ^a^ | 86.61 ^a^ |  | 0.30 (0.23, 0.41) ^a^ | 95.13 ^a^ |  | 0.33 (0.23, 0.43) ^a^ | 93.81 |
|  |  | Model 2 | 0.31 (0.23, 0.44) ^bd^ | 83.93 ^bd^ |  | 0.33 (0.24, 0.43) ^bd^ | 95.13 ^b^ |  | 0.35 (0.24, 0.45) ^d^ | 92.04 |
|  |  | Model 3 | 0.36 (0.27, 0.48) ^cef^ | 77.68 ^ef^ |  | 0.36 (0.27, 0.49) ^cef^ | 89.38 ^ef^ |  | 0.38 (0.26, 0.50) ^cef^ | 87.17 ^cef^ |
|  | Scenario 2 | Model 4 | 0.38 (0.30, 0.51) ^g^ | 73.21 ^g^ |  | 0.38 (0.31, 0.51) ^g^ | 89.38 |  | 0.39 (0.31, 0.51) ^g^ | 88.50 ^g^ |
|  |  | Model 5 | 0.42 (0.34, 0.54) ^hi^ | 67.86 ^hi^ |  | 0.42 (0.35, 0.53) ^hi^ | 86.28 ^hi^ |  | 0.44 (0.36, 0.55) ^hi^ | 84.51 ^hi^ |
| **Vitamin B_2_**  **(mg/d)** | Before simulation | | 0.53 (0.38, 0.72) | 44.20 |  | 0.52 (0.36, 0.73) | 59.73 |  | 0.52 (0.35, 0.75) | 58.85 |
|  | Scenario 1 | Model 1 | 0.36 (0.27, 0.47) ^a^ | 80.36 ^a^ |  | 0.37 (0.28, 0.52) ^a^ | 88.94 ^a^ |  | 0.40 (0.30, 0.52) ^a^ | 80.53 ^a^ |
|  |  | Model 2 | 0.60 (0.42, 0.78) ^bd^ | 35.71 ^bd^ |  | 0.59 (0.44, 0.81) ^bd^ | 52.21 ^bd^ |  | 0.57 (0.42, 0.84) ^bd^ | 51.77 ^bd^ |
|  |  | Model 3 | 0.55 (0.40, 0.70) ^cef^ | 41.07 ^ef^ |  | 0.54 (0.42, 0.75) ^cef^ | 58.41 ^ef^ |  | 0.53 (0.39, 0.76) ^cef^ | 59.73 ^ef^ |
|  | Scenario 2 | Model 4 | 0.77 (0.66, 0.89) ^g^ | 3.57 ^g^ |  | 0.76 (0.67, 0.94) ^g^ | 12.83 ^g^ |  | 0.82 (0.72, 0.98) ^g^ | 11.06 ^g^ |
|  |  | Model 5 | 0.73 (0.63, 0.86) ^hi^ | 5.80 ^h^ |  | 0.72 (0.62, 0.91) ^hi^ | 20.80 ^hi^ |  | 0.77 (0.67, 0.95) ^hi^ | 20.35 ^hi^ |
| **Vitamin B_3_**  **(mgNE/d)** | Before simulation | | 6.28 (4.90, 8.57) | 26.34 |  | 6.59 (5.19, 9.36) | 47.79 |  | 7.69 (5.43, 10.22) | 36.28 |
|  | Scenario 1 | Model 1 | 6.33 (4.99, 8.66) ^a^ | 25.45 |  | 6.76 (5.28, 9.50) ^a^ | 47.35 |  | 7.81 (5.43, 10.28) ^a^ | 35.40 |
|  |  | Model 2 | 6.27 (4.91, 8.67) ^bd^ | 26.79 |  | 6.62 (5.24, 9.38) ^d^ | 47.79 |  | 7.67 (5.40, 10.17) ^d^ | 36.73 |
|  |  | Model 3 | 6.80 (5.32, 9.35) ^cef^ | 20.54 ^cef^ |  | 7.28 (5.54, 10.15) ^cef^ | 40.27 ^cef^ |  | 8.05 (5.75, 10.66) ^cef^ | 33.19 ^cf^ |
|  | Scenario 2 | Model 4 | 6.49 (5.08, 8.72) ^g^ | 23.66 ^g^ |  | 6.73 (5.22, 9.40) ^g^ | 44.25 ^g^ |  | 7.91 (5.59, 10.40) ^g^ | 33.63 ^g^ |
|  |  | Model 5 | 7.00 (5.45, 9.14) ^hi^ | 20.09 ^hi^ |  | 7.22 (5.74, 10.00) ^hi^ | 34.96 ^hi^ |  | 8.38 (6.19, 10.86) ^hi^ | 28.32 ^hi^ |
| **Vitamin B_6_**  **(mg/d)** | Before simulation | | 0.67 (0.50, 0.90) | 24.55 |  | 0.70 (0.52, 0.93) | 36.73 |  | 0.75 (0.50, 1.01) | 33.19 |
|  | Scenario 1 | Model 1 | 0.64 (0.47, 0.87) ^a^ | 29.91 ^a^ |  | 0.69 (0.51, 0.89) ^a^ | 40.71 ^a^ |  | 0.74 (0.50, 0.97) ^a^ | 34.51 |
|  |  | Model 2 | 0.67 (0.51, 0.90) ^bd^ | 24.55 ^d^ |  | 0.71 (0.54, 0.93) ^bd^ | 37.17 ^d^ |  | 0.76 (0.52, 1.01) ^bd^ | 32.74 |
|  |  | Model 3 | 0.68 (0.51, 0.91) ^cef^ | 23.66 ^e^ |  | 0.72 (0.55, 0.94) ^cef^ | 34.51 ^ef^ |  | 0.77 (0.53, 1.01) ^cef^ | 31.42 ^e^ |
|  | Scenario 2 | Model 4 | 0.73 (0.56, 0.94) ^g^ | 17.41 ^g^ |  | 0.76 (0.58, 0.97) ^g^ | 27.43 ^g^ |  | 0.83 (0.59, 1.05) ^g^ | 26.11 ^g^ |
|  |  | Model 5 | 0.73 (0.56, 0.96) ^hi^ | 15.63 ^h^ |  | 0.77 (0.59, 0.97) ^hi^ | 26.11 ^h^ |  | 0.84 (0.60, 1.07) ^hi^ | 24.78 ^h^ |
| **Vitamin B_9_**  **(μgDFE/d)** | Before simulation | | 112.54 (81.38, 156.13) | 62.95 |  | 114.06 (83.30, 156.23) | 73.01 |  | 122.35 (91.04, 158.69) | 70.35 |
|  | Scenario 1 | Model 1 | 187.19 (141.40, 240.13) ^a^ | 22.32 ^a^ |  | 180.11 (132.91, 242.16) ^a^ | 32.74 ^a^ |  | 181.85 (128.96, 245.04) ^a^ | 34.51 ^a^ |
|  |  | Model 2 | 114.62 (82.43, 158.32) ^bd^ | 62.50 ^d^ |  | 116.80 (86.44, 157.82) ^bd^ | 70.80 ^d^ |  | 123.44 (92.61, 166.55) ^bd^ | 68.14 ^d^ |
|  |  | Model 3 | 134.06 (102.04, 179.11) ^cef^ | 47.77 ^cef^ |  | 134.40 (100.87, 183.12) ^cef^ | 61.50 ^cef^ |  | 139.66 (104.01, 181.11) ^cef^ | 57.08 ^cef^ |
|  | Scenario 2 | Model 4 | 121.47 (90.89, 167.11) ^g^ | 59.38 ^g^ |  | 122.40 (92.96, 164.50) ^g^ | 69.47 ^g^ |  | 132.87 (101.06, 169.15) ^g^ | 65.04 ^g^ |
|  |  | Model 5 | 138.93 (108.64, 183.27) ^hi^ | 43.30 ^hi^ |  | 139.13 (112.84, 185.16) ^hi^ | 58.85 ^hi^ |  | 154.23 (120.59, 192.01) ^hi^ | 45.58 ^hi^ |
| **Vitamin B_12_**  **(μg/d)** | Before simulation | | 1.53 (1.01, 2.30) | 16.52 |  | 1.65 (1.02, 2.63) | 24.55 |  | 1.68 (1.07, 2.90) | 21.43 |
|  | Scenario 1 | Model 1 | 1.16 (0.81, 1.89) ^a^ | 24.11 ^a^ |  | 1.31 (0.92, 2.20) ^a^ | 31.70 ^a^ |  | 1.34 (0.95, 2.45) ^a^ | 27.68 ^a^ |
|  |  | Model 2 | 1.41 (1.00, 2.14) ^bd^ | 14.29 ^d^ |  | 1.83 (1.13, 2.77) ^bd^ | 19.91 ^bd^ |  | 1.62 (1.04, 2.96) ^d^ | 20.80 ^d^ |
|  |  | Model 3 | 1.85 (1.21, 2.73) ^cef^ | 9.38 ^cef^ |  | 1.95 (1.29, 2.88) ^cef^ | 14.73 ^cef^ |  | 1.84 (1.28, 3.14) ^cef^ | 13.84 ^cef^ |
|  | Scenario 2 | Model 4 | 1.85 (1.44, 2.55) ^g^ | 1.34 ^g^ |  | 2.04 (1.49, 2.95) ^g^ | 3.54 ^g^ |  | 2.03 (1.53, 3.30) ^g^ | 4.87 ^g^ |
|  |  | Model 5 | 2.21 (1.83, 2.80) ^hi^ | 0.00 ^h^ |  | 2.38 (1.94, 3.17) ^hi^ | 0.00 ^hi^ |  | 2.47 (2.04, 3.50) ^hi^ | 0.88 ^hi^ |
| **Vitamin C**  **(mg/d)** | Before simulation | | 43.81 (24.67, 69.70) | 37.95 |  | 40.93 (22.56, 63.17) | 48.67 |  | 42.22 (21.70, 77.33) | 47.35 |
|  | Scenario 1 | Model 1 | 36.21 (19.19, 62.07) ^a^ | 47.32 ^a^ |  | 36.11 (19.80, 57.20) ^a^ | 54.42 ^a^ |  | 38.58 (20.07, 72.18) ^a^ | 50.88 ^a^ |
|  |  | Model 2 | 37.93 (20.88, 64.18) ^bd^ | 46.43 ^b^ |  | 37.90 (20.25, 59.49) ^bd^ | 52.65 ^b^ |  | 40.35 (21.36, 76.60) ^d^ | 48.67 |
|  |  | Model 3 | 43.40 (27.30, 70.74) ^cef^ | 37.50 ^ef^ |  | 43.87 (23.75, 65.94) ^cef^ | 45.13 ^ef^ |  | 47.11 (25.04, 84.29) ^cef^ | 45.58 ^ef^ |
|  | Scenario 2 | Model 4 | 45.02 (27.02, 71.45) ^g^ | 36.61 |  | 42.54 (24.07, 64.18) ^g^ | 47.79 |  | 44.00 (23.68, 79.59) ^g^ | 47.35 |
|  |  | Model 5 | 50.60 (30.95, 77.03) ^hi^ | 30.80 ^hi^ |  | 47.12 (31.11, 70.84) ^hi^ | 40.27 ^hi^ |  | 49.67 (29.97, 83.11) ^hi^ | 42.04 ^hi^ |
| **Vitamin D**  **(μg/d)** | Before simulation | | 0.03 (0.00, 2.23) | 96.88 |  | 0.00 (0.00, 1.65) | 99.56 |  | 0.00 (0.00, 0.55) | 99.12 |
|  | Scenario 1 | Model 1 | 0.00 (0.00, 0.00) ^a^ | 98.21 |  | 0.00 (0.00, 0.00) ^a^ | 100.00 |  | 0.00 (0.00, 0.00) ^a^ | 99.12 |
|  |  | Model 2 | 0.00 (0.00, 0.00) ^b^ | 98.21 |  | 0.00 (0.00, 0.00) ^bd^ | 100.00 |  | 0.00 (0.00, 0.00) ^b^ | 99.12 |
|  |  | Model 3 | 1.41 (0.71, 2.16) ^cef^ | 97.32 |  | 1.32 (0.70, 1.83) ^cef^ | 100.00 |  | 1.00 (0.49, 1.77) ^cef^ | 98.67 |
|  | Scenario 2 | Model 4 | 0.03 (0.00, 2.23) ^g^ | 96.88 |  | 0.00 (0.00, 1.65) ^g^ | 99.56 |  | 0.00 (0.00, 0.55) ^g^ | 99.12 |
|  |  | Model 5 | 2.32 (1.65, 3.31) ^hi^ | 96.88 |  | 2.19 (1.31, 2.79) ^hi^ | 99.56 |  | 1.92 (1.33, 2.48) ^hi^ | 98.67 |

The intakes of vitamins below the estimated average requirement were perceived as inadequate.

Wilcoxon matched-pairs signed rank test and McNemar paired Chi-square test were used to compare the differences in nutrient intake and the changes in the proportion of preschool children with inadequate nutrient intake before and after modeling, respectively.

^a^: Model 1 *vs.* Before simulation *P* < 0.05; ^b^: Model 2 *vs.* Before simulation *P* < 0.05; ^c^: Model 3 *vs.* Before simulation *P* < 0.05; ^d^: Model 2 *vs.* Model 1 *P* < 0.05; ^e^: Model 3 *vs.* Model 1 *P* < 0.05; ^f^: Model 3 *vs.* Model 2 *P* < 0.05; ^g^: Model 4 *vs.* Before simulation *P* < 0.05; ^h^: Model 5 *vs.* Before simulation *P* < 0.05; ^i^: Model 5 *vs.* Model 4 *P* < 0.05.

Model 1: The intake of liquid milk equivalents was replaced by soymilk at a matching volume.

Model 2: The intake of liquid milk equivalents was replaced by cow’s milk at a matching volume.

Model 3: The intake of liquid milk equivalents was replaced by FMP-PSC at a matching volume.

Model 4: The amount of cow’s milk was added to make the dairy intake of each child reach the recommended amount.

Model 5: The amount of FMP-PSC was added to make the dairy intake of each child reach the recommended amount.
